# Tuberculosis treatment outcomes after transfer or release from incarceration: a retrospective cohort study from Brazil

**DOI:** 10.1186/s44263-025-00210-5

**Published:** 2025-10-21

**Authors:** Yasmine Mabene, José Victor Bortolotto Bampi, Everton Ferreira Lemos, Roberto de Oliveira, Crhistinne Gonçalves, Maria de Lourdes Delgado Alves, Maridiane Coutinho Echevarria, Julio Croda, Jason R. Andrews, Yiran E. Liu

**Affiliations:** 1 Division of Infectious Diseases and Geographic Medicine, Department of Medicine, Stanford University, Stanford, CA USA; 2 Federal University of Mato Grosso do Sul, Infectious and Parasitic Diseases Program, Campo Grande, Mato Grosso do Sul Brazil; 3 State University of Mato Grosso do Sul, Dourados, Mato Grosso do Sul Brazil; 4 Fundação Oswaldo Cruz, Mato Grosso do Sul, Campo Grande, Mato Grosso do Sul Brazil; 5 Post Graduate Program in Health Sciences, Federal University of Grande Dourados, Dourados, Mato Grosso do Sul Brazil; 6 School of Medicine, Federal University of Mato Grosso do Sul, Campo Grande, Mato Grosso do Sul Brazil; 7 Mato Grosso do Sul State Secretariat of Health, Campo Grande, Mato Grosso do Sul Brazil; 8 Directorate of Penitentiary Assistance - State Agency of Administration of the Penitentiary System, Campo Grande, Mato Grosso do Sul Brazil; 9 Yale University, Epidemiology of Microbial Diseases, New Haven, CT USA; 10 SEICHE Center for Health and Justice, Yale School of Medicine and Yale Law School, New Haven, CT USA

**Keywords:** Incarceration, Prisons, Tuberculosis, Treatment, Cascade of care, Linkage to care, Continuity of care, Persons deprived of liberty, Brazil

## Abstract

**Background:**

Tuberculosis (TB) disproportionately affects people deprived of liberty (PDL). Prior studies have shown higher TB treatment completion rates among PDL compared to the general population. However, little is known about how incarceration-related movements such as transfers between facilities and releases to the community affect TB treatment outcomes.

**Methods:**

We linked person-level incarceration data with TB notifications data from the Notifiable Disease Information System for the Brazilian state of Mato Grosso do Sul between January 2006 and December 2018. We constructed a cohort of PDL who were newly diagnosed with drug-susceptible TB and initiated treatment. We compared treatment outcomes between individuals who remained in the same carceral facility and those who were transferred to other facilities or released from incarceration during treatment. We computed the covariate-adjusted relative risk of unfavorable treatment outcomes for individuals transferred or released during treatment.

**Results:**

We identified 1261 PDL who initiated TB treatment. Of these individuals, 842 (66.8%) remained in the same carceral facility, 256 (20.3%) were transferred to other facilities, and 163 (12.9%) were released to the community during treatment. Among those who remained in the same carceral facility, 72.9% (614/842) were successfully treated within 8 months following treatment initiation. In contrast, only 61.7% (158/256) of those who were transferred and 50.3% (82/163) of those who were released achieved TB treatment success within 8 months. After adjusting for covariates, the risk of unfavorable treatment outcomes was 1.4 (95% *CI*: 1.2 to 1.7) times as high for individuals transferred to other facilities and 1.6 (95% *CI*: 1.3 to 2.0) times as high for individuals released from incarceration, compared to those who remained in the same facility during treatment. For individuals released less than 2 months into treatment, the risk of unfavorable treatment outcomes was twice as high (adjusted relative risk [aRR]: 2.1, 95% *CI*: 1.6–2.7).

**Conclusions:**

Transfers between facilities and releases from incarceration are common and may pose barriers to TB treatment completion. Strategies for ensuring continuity of care across carceral facilities and between prison and community health systems are urgently needed to improve TB outcomes for individuals impacted by incarceration.

**Supplementary Information:**

The online version contains supplementary material available at 10.1186/s44263-025-00210-5.

## Background

In 2023, 10.8 million people fell ill with tuberculosis (TB) worldwide, and 1.25 million people died from TB [1]. Persons deprived of liberty (PDL) face an increased risk of TB compared to the general population due to factors such as prison overcrowding, poor ventilation, undernutrition, and limited healthcare access [2, 3]. High rates of TB in prisons can also spill over into communities through prison staff, visitors, and individuals released from incarceration [4, 5]. Targeted efforts to address TB in prisons are needed to reduce TB morbidity and mortality for PDL and to progress toward population-wide TB elimination.

In 2023, the United Nations High-Level Meeting established global targets aimed at ensuring a 90% treatment completion rate for all people with TB [6]. Achieving these targets is especially crucial for vulnerable populations, including PDL. Although previous studies have reported higher TB treatment completion rates for PDL compared to the general population, these rates still fall short of the UN’s 90% target [7–12]. More research is needed to identify barriers and facilitators to treatment completion among PDL to improve TB outcomes in this population.

PDL who begin TB treatment may face interruptions to care following transfers between carceral facilities or release from incarceration. Research has shown this for PDL living with HIV, finding that releases from jails and prisons may disrupt the HIV continuum of care [13–15]. Similar challenges have been observed among people undergoing treatment for substance use disorders, including low rates of treatment retention post-release [16, 17]. Studies in the USA on TB preventive therapy for people with latent tuberculosis infection (LTBI) have found lower completion rates for individuals released during treatment, with some studies reporting rates as low as 20% [18–20]. Far less is known about how carceral movements impact treatment continuity and outcomes for individuals with active TB. Studies in Uganda and Zambia found treatment disruptions and increased rates of loss to follow-up for PDL who were transferred or released during treatment, as well as for those residing in prisons with shorter incarceration sentences [21–23]. However, there remains a significant knowledge gap regarding TB treatment outcomes and linkage to care among individuals experiencing carceral movements in other regions and settings.

In Brazil, incarceration is the leading driver of the TB epidemic, accounting for approximately 37% of new cases [24]. Brazil, which has the third largest prison population in the world, has the highest number of PDL developing TB each year [25, 26]. Despite this, carceral movements for individuals diagnosed with TB in Brazil are not well documented, and the impact of carceral movements on TB treatment in Brazil remains unknown. PDL in Brazil may reside in closed prisons, semi-open prisons (where individuals leave prison during the day for work and return at night), or in police detention. Varying frequencies of movements and healthcare resources across carceral facilities may affect treatment outcomes and continuity of care. As both TB incidence and incarceration rates rise in Brazil, it is essential to investigate the potential effects of carceral movements on continuity of care to inform strategies for improving TB treatment outcomes for PDL.

In this study, we link individual-level incarceration and TB notifications data for the Brazilian state of Mato Grosso do Sul to assess the impact of carceral movements on TB treatment outcomes. We examine the proportion of individuals experiencing transfers or releases during TB treatment and determine the relative risk of unfavorable treatment outcomes compared to individuals who remain in the same carceral facility during treatment. We focus our analysis on Mato Grosso do Sul, where study authors are based and data were available. A Portuguese translation of the abstract is provided in Supplementary material 1.

## Methods

### Study design

We conducted a retrospective cohort study, using previously collected administrative data, of PDL who were newly diagnosed with drug-sensitive TB (pulmonary and extra-pulmonary) and initiated treatment in Mato Grosso do Sul state, Brazil, between January 1, 2006, and June 30, 2018, with follow-up of TB outcomes through June 30, 2020. We use 2018 for the final year of inclusion in our analysis due to the availability of incarceration movement data through December 31, 2018, but we access TB notification data through June 30, 2020, to include 2-year outcomes. We examined TB treatment outcomes among individuals who were transferred to other carceral facilities and/or released from incarceration during the 6-month treatment period, compared to those remaining in the same carceral facility. We included PDL who resided in closed prisons, semi-open prisons, or police detention at the time of treatment initiation. We excluded individuals with prior TB diagnoses, those with drug resistance at the time of diagnosis or changes in diagnosis or treatment regimen, and those under the age of 18 (Supplementary material 2: Text S1). Individuals may have a history of incarceration but no history of TB within our study period. All data in this study were stored on secure encrypted devices and servers approved for high-risk data. Following data linkage, identifiers were removed for remaining analyses.

### Data sources

We accessed statewide individual-level data on TB notifications and treatment outcomes between January 1, 2006, and June 30, 2020, from *Sistema de Informação de Agravos de Notificação* (SINAN). To determine incarceration status and movements during and after TB treatment initiation, we linked SINAN with Sistema Integrado de Gestão Operacional (SIGO), a database containing movements within the carceral system (i.e., entries, transfers, and releases) in Mato Grosso do Sul from January 1, 2006, to June 30, 2018. To account for incomplete data on deaths during the follow-up period, we performed additional linkage with the mortality database, Sistema de Informações Sobre Mortalidade (SIM), accessed between January 1, 2006, and June 30, 2020.

### Data processing and linkage

For the incarceration (SIGO) and TB (SINAN) databases, where an individual could have multiple distinct entries (corresponding to multiple carceral movements, TB diagnoses, etc.), we identified records belonging to unique individuals using approximate string matching over name and mother’s name, with additional comparisons of identification numbers and dates of birth when available (Supplementary material 2: Text S1). Approximate string matching was performed using stringdist in R version 4.4.1 [27, 28].

We linked unique individuals across the incarceration and TB databases using approximate string matching on shared identifiers (name and mother’s name), resulting in a sensitivity of 90% and specificity of 95% (Supplementary material 2: Text S1). We performed subsequent filtering steps to exclude false-positive matches (Supplementary material 2: Text S1). Among matched individuals, we then searched for deaths within the mortality (SIM) database using name, mother’s name, and date of birth. Although the TB (SINAN) database includes information on deaths, we additionally matched individuals to the mortality database (SIM) to account for unreported deaths (Supplementary material 2: Text S1). Exact criteria for database linkage can be found in Supplementary material 2: Fig. S1.

### Group assignment and outcome determination

We categorized individuals by whether they experienced carceral movements (transfers to other facilities or release from incarceration) within 6 months following treatment initiation. Each individual was assigned to one of three groups: (1) individuals who remained in the same carceral facility throughout the treatment period (the “stationary” group), (2) individuals who were transferred to another carceral facility at least once but were not released (the “transferred” group), and (3) individuals who were released from incarceration (the “released” group; this group may also include individuals who escaped incarceration). To avoid exposure misclassification, only movements that occurred prior to ascertainment of treatment outcomes were considered in group assignment (Supplementary materiale 2: Text S1). In an additional analysis, we stratified the released group into two subgroups based on the timing of release (released within or after 2 months following treatment initiation). This was done to examine whether treatment outcomes varied by timing of release, as treatment interruptions during the first 2 months (the “intensive phase” of TB treatment) may more seriously impact outcomes compared to interruptions later on, during the “continuation phase” of TB treatment [29].

In the primary analysis, we evaluated TB treatment outcomes at 8-month posttreatment initiation. While treatment completion takes 6 months, we used the 8-month point to account for delays in reporting treatment outcomes. We additionally assessed treatment outcomes at 2-year posttreatment initiation. This longer follow-up allowed us to capture outcomes for cases where individuals may have discontinued and later restarted treatment, as well as to identify deaths or other outcomes that were reported after the initial 8-month period. All outcomes in the SINAN database are closed after 2-year posttreatment initiation. We considered a treatment outcome to be positive if the individual achieved treatment success; all other outcomes were considered unfavorable treatment outcomes. We categorized treatment outcomes in our study using the SINAN guidelines as follows [30]:

1. *Treatment success*: Patients who obtained two negative sputum smears (one at any follow-up month and one at the end of treatment) or those who completed treatment without evidence of treatment failure and were discharged based on clinical and radiological criteria, in accordance with Brazilian guidelines [30]
2. *No case status update (primary)*: Patients whose case had no updates in the TB (SINAN) database following treatment initiation
3. *No case status update (after resuming treatment after treatment discontinuation or referral to different health facility)*: Patients who discontinued treatment or transferred health facilities and were later linked to care (resumed treatment) without further case updates in the TB (SINAN) database
4. *Treatment discontinuation with no follow-up record*: Patients who interrupted treatment for 30 or more consecutive days and had no subsequent updates in the TB (SINAN) database
5. *Transferred health facility with no follow-up record*: Patients who were transferred to another health facility and had no subsequent updates in the TB (SINAN) database
6. *Drug-resistant TB (DR-TB)*: Patients with confirmed resistance to any antituberculosis drug after treatment initiation
7. *Death (TB)*: Patients with tuberculosis as the cause of death
8. *Death (non-TB)*: Patients with cause of death other than tuberculosis
9. *Change of diagnosis/regimen*: Patients whose initial TB diagnosis was incorrect or patients who changed treatment regimen due to drug intolerance or toxicity
10. *Treatment failure*: Patients must meet one of the following conditions — persistent positive sputum smear microscopy at end of treatment, patients who began treatment with a strongly positive bacilloscopy result and remained positive until the fourth month of treatment, an initial positive bacilloscopy result followed by negative results, and then a return to positive results for two consecutive months, starting from the fourth month of treatment
11. *Unknown*: Patients whose outcomes were recorded as not available (N/A) in SINAN

Note, we classify no case update in SINAN at 8 months as an unfavorable outcome. As standard TB treatment typically lasts 6 months, the absence of an update in SINAN by 8-month post-notification likely reflects disengagement from care and/or lack of timely follow-up and case management by the health sector. Additional information on this outcome can be found in Supplementary material 2: Text S2. We consider individuals to be lost to follow-up if they either discontinued treatment or were referred to other health facilities with no follow-up record.

### Statistical analysis

We used the chi-square test to compare characteristics across groups and to assess differences in the proportion of treatment success across carceral movement groups. To determine the effect of carceral movements on treatment outcomes, we performed multivariable Poisson regression, adjusting for race, age, education, alcohol use disorder, mental illness, incarceration history in the past 2 years (total duration and unique entries into the carceral system), and facility type at treatment initiation (closed prison, semi-open prison, police detention) [31]. We included facility type at treatment initiation as a covariate due to variation across carceral facilities in healthcare, security level, and movements. Covariates with less than 25% missingness were imputed using missForest version 1.5 in R (Supplementary material 2: Table S1) [32]. For covariates with greater missingness, we retained “unknown/missing” as a level in the regression. For the regression analysis, we restricted our cohort to individuals who initiated TB treatment after January 1, 2007. This enabled adjustment for incarceration history in the past 2 years.

We conducted two sensitivity analyses for the regression models. First, we excluded from the regression individuals who died from non-TB causes within the first 6 months following treatment initiation. This exclusion was made to mitigate potential survival-induced non-exchangeability bias, as individuals in the transferred or released group had to survive long enough to experience carceral movements. In a second sensitivity analysis, we adjusted for individual carceral facility in the regression model to account for potential differences in healthcare across facilities.

## Results

### Baseline cohort characteristics

**Fig. 1:**
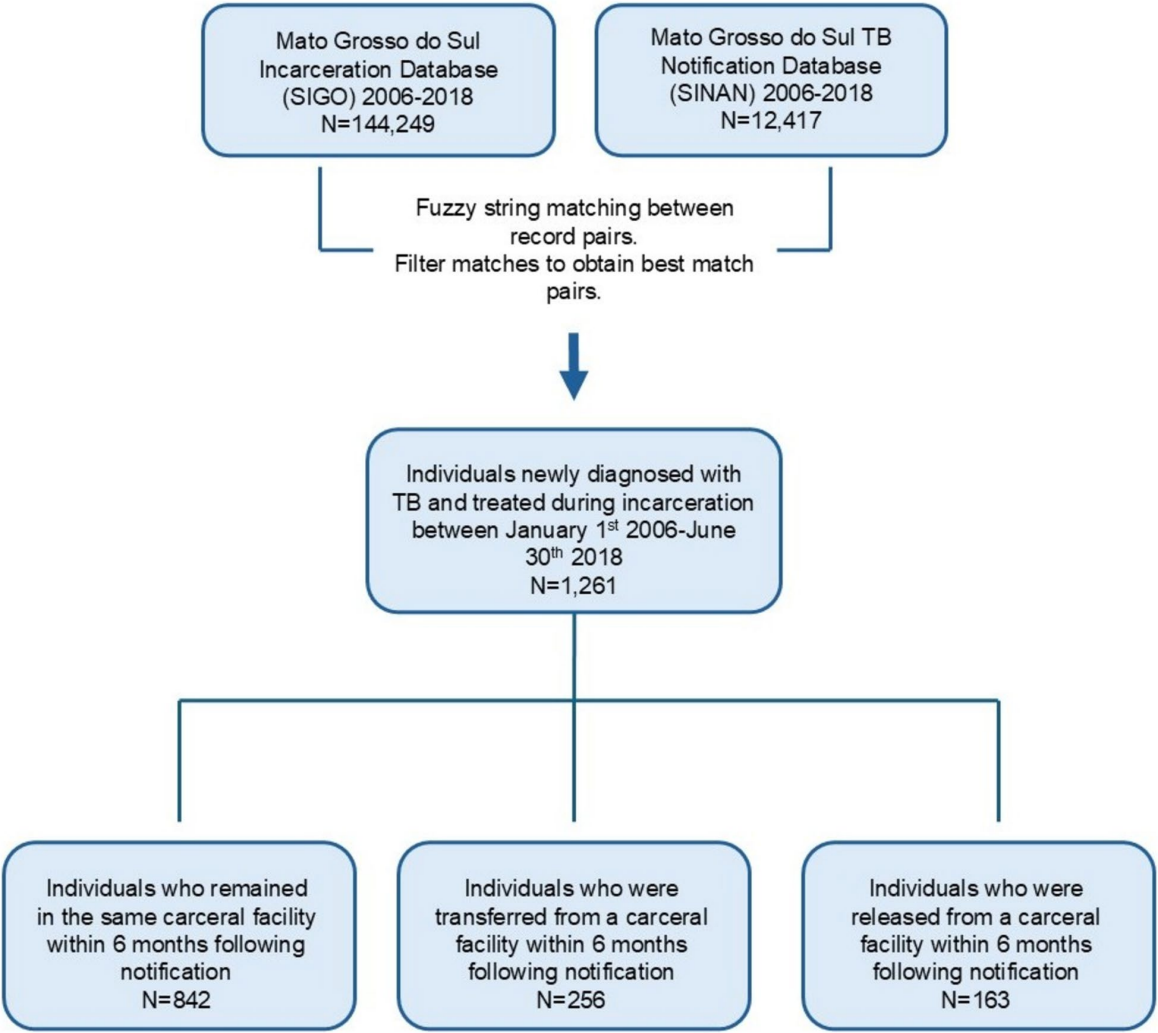
Database linkage between SIGO and SINAN. Identification of individuals who were diagnosed with TB and initiated TB treatment during incarceration and classification by carceral movements during treatment. See Supplementary material 2: Text S1 for matching and inclusion criteria. SIGO, Sistema Integrado de Gestão Operacional (incarceration database); SINAN, Sistema de Informação de Agravos de Notificação (TB notifications database)

After database linkage, we obtained a cohort of 1261 PDL who initiated TB treatment between January 1, 2006, and June 30, 2018 (Fig. 1). Our cohort primarily consisted of male individuals under the age of 30 (Table 1). Nearly half of the participants were Black or mixed race (*N* = 578, 45.8%), and most individuals (*N* = 685, 54.3%) indicated not having completed high school. Additionally, 80 (6.3%) individuals reported having alcohol use disorder, and less than 1% (9, 0.7%) were reported to have mental illness. Sixty-eight (5.4%) individuals tested positive for HIV, and 351 (27.8%) had no testing reported. Eighteen (1.4%) individuals reported having diabetes, and 290 (23.0%) had no data reported. Most individuals (944, 74.9%) resided in closed prisons at the time of treatment initiation (Table 1). In the 2 years preceding treatment initiation, 1014 (80.4%) had been in custody for more than 1 year, and approximately 1 in 4 individuals (328, 26.0%) had experienced multiple incarcerations. The median time spent incarcerated was 23 (*IQR*: 15–23) months, and the median number of distinct incarcerations was 1 (*IQR*: 1–2).

**Table 1:** Demographic and clinical characteristics of individuals grouped by carceral movement during TB treatment

Characteristics	Categories	Stationary *N* = 842	Transfer *N* = 256	Release *N* = 163	*p*-value
**Facility type**	Closed prison	654 (77.7%)	204 (79.7%)	86 (52.8%)	< 0.001
	Semi-open prison	99 (11.8%)	29 (11.3%)	56 (344%)	
	Police detention	89 (10.6%)	23 (9.0%)	21 (12.9%)	
**Sex**	Male	820 (97.4%)	251 (98.0%)	155 (95.1%)	0.16
	Female	22 (2.6%)	5 (2.0%)	8 (4.9%)	
**Age**	18–29	415 (49.3%)	146 (57.0%)	75 (46.0%)	0.15
	30–44	332 (39.4%)	93 (36.3%)	67 (41.1%)	
	> 45	92 (10.9%)	17 (6.6%)	20 (12.3%)	
	Not reported	3 (0.4%)	0 (0.0%)	1 (0.6%)	
**Race**	White	278 (33.0%)	82 (32.0%)	48 (29.4%)	0.76
	Mixed race	328 (39.0%)	92 (35.9%)	72 (44.2%)	
	Black	59 (7.0%)	17 (6.6%)	10 (6.1%)	
	Asian	12 (1.4%)	4 (1.6%)	1 (0.6%)	
	Indigenous	15 (1.8%)	8 (3.1%)	5 (3.1%)	
	Not reported	150 (17.8%)	53 (20.7%)	27 (16.6%)	
**Education**	Did not complete high school	449 (53.3%)	151 (59.0%)	85 (52.1%)	0.08
	Completed high school or more	28 (3.3%)	9 (3.5%)	12 (7.4%)	
	Not reported	365 (43.3%)	96 (37.5%)	66 (40.5%)	
**Alcohol use disorder**	No	591 (70.2%)	181 (70.7%)	108 (66.3%)	0.41
	Yes	49 (5.8%)	15 (5.9%)	16 (9.8%)	
	Not reported	202 (24.0%)	60 (23.4%)	39 (23.9%)	
**Mental illness**	No	643 (76.4%)	201 (78.5%)	119 (73.0%)	0.32
	Yes	5 (0.6%)	1 (0.4%)	3 (1.8%)	
	Not reported	194 (23.0%)	54 (21.1%)	41 (25.2%)	
**Total time incarcerated in the past 2 years**	< 1 year	153 (18.2%)	44 (17.2%)	50 (30.7%)	< 0.001
	1–2 years	689 (81.8%)	212 (82.8%)	113 (69.3%)	
**Number of distinct incarcerations**^a^** in the past 2 years**	1	653 (77.6%)	167 (65.2%)	113 (69.3%)	< 0.001
	> 1	189 (22.4%)	89 (34.8%)	50 (30.7%)	

Facility type refers to location at the time of TB notification.

^a^The study was approved by the institutional review boards (IRBs) of Federal University of Mato Grosso do Sul (UFMS) (#7.060.054) and Stanford University (#50466). We only accessed routinely collected data. All data in this study were stored on secure encrypted devices and servers approved for high-risk data. This study conformed to the principles of the Helsinki Declaration.

Most individuals in the cohort (842, 66.7%) remained in the same carceral facility in the 6 months following TB treatment initiation. Another 256 (20.3%) individuals were transferred to other carceral facilities, and 163 (12.9%) were released to the community within 6 months of treatment initiation (Table 1). Individuals who initiated treatment in semi-open prisons were more likely to be released to the community during treatment compared to those who initiated treatment in any other facility type (Supplementary material 2: Fig. S2). At the time of treatment initiation, a higher proportion of individuals in the released group had spent less than one of the past 2 years incarcerated (30.7%), compared to individuals in the stationary (18.2%) and transferred (17.2%) groups (*p*-value < 0.001) (Table 1). Lastly, the stationary group had a lower proportion of individuals with multiple prior incarcerations in the last 2 years (22.4%), compared to the transferred (34.8%) and released (30.7%) groups (*p*-value < 0.001). The distributions of the year of TB notification and carceral facility at the time of treatment initiation can be found in Supplementary material 2: Table S2 and Supplementary material 2: Table S3.

### TB treatment outcomes by carceral movement group

Across the entire cohort, 854 (67.7%) of individuals successfully completed treatment within 8 months following treatment initiation. Another 179 (14.2%) individuals were lost to follow up, 66 (5.2%) had no case status updates, and 38 (3.0%) died (Table 2). Among those who remained in the same carceral facility in the 6 months following TB notification, 614 (72.9%) had successful treatment, 84 (10.0%) were lost to follow-up, 30 (3.6%) had no case status updates, and 33 (3.9%) died. Compared to those who remained in the same carceral facility, those who experienced movements during treatment had significantly lower treatment success rates: 61.7% (*N* = 158) in the transferred group and 50.3% (*N* = 82) in the released group (*p*-value < 0.001). At 2 years following treatment initiation, success rates remained lower for those transferred (65.8%, *N* = 168) or released (54.6%, *N* = 89) compared to those who remained stationary (74.3%, *N* = 626) (*p*-value < 0.001) (Supplementary material 2: Table S4). Additionally, the released group was less likely to have record closure in SINAN within 8 months of notification, with 12.3% missing case status updates compared to only 3.6% in the stationary group (*p*-value < 0.001), suggesting greater care disengagement in this group.

**Table 2:** TB treatment outcomes 8 months following treatment initiation

Treatment outcome	Stationary group (*N* = 842)	Transferred group (*N* = 256)	Released group (*N* = 163)
Treatment success	614 (72.9%)	158 (61.7%)	82 (50.3%)
No case status update (primary)	13 (1.5%)	4 (1.6%)	11 (6.7%)
No case update (after resuming treatment after treatment discontinuation or referral to different health facility)	17 (2.0%)	12 (4.7%)	9 (5.5%)
Referred to different health facility with no follow-up record	26 (3.1%)	30 (11.7%)	11 (6.7%)
Treatment discontinuation with no follow-up record	58 (6.9%)	25 (9.8%)	29 (17.8%)
Death TB	12 (1.4%)	1 (0.4%)	1 (0.6%)
Death non-TB	21 (2.5%)	1 (0.4%)	2 (1.2%)
DR-TB	1 (0.1%)	1 (0.4%)	1 (0.6%)
Unknown	80 (9.5%)	24 (9.4%)	17 (10.4%)

Carceral movement groups were determined based on movements occurring up to 6 months following treatment initiation. Individuals may have experienced additional carceral movements before the end of the follow-up period, but these movements were not considered for group assignment. Treatment failure is not included within this table as no patients in the cohort experienced treatment failure. Patients with a change of diagnosis/regimen were excluded from the analysis. Percentages may not add up to 100 due to rounding

The proportion of individuals who were lost to follow-up after being referred to a different health facility was over three times as high for individuals who were transferred to other carceral facilities (11.7%) compared to those who remained in the same location (3.1%) (*p*-value < 0.001). For individuals who were released, this proportion was more than twice as high (6.7%) (*p*-value = 0.04). Among those released from carceral facilities who were referred to another health facility or experienced treatment discontinuation within 8 months of notification, 18 (36.7%) were subsequently linked to care, and 10 (20.4%) successfully completed treatment within the state over a 2-year period.

### Factors associated with unfavorable treatment outcomes

**Fig. 2:**
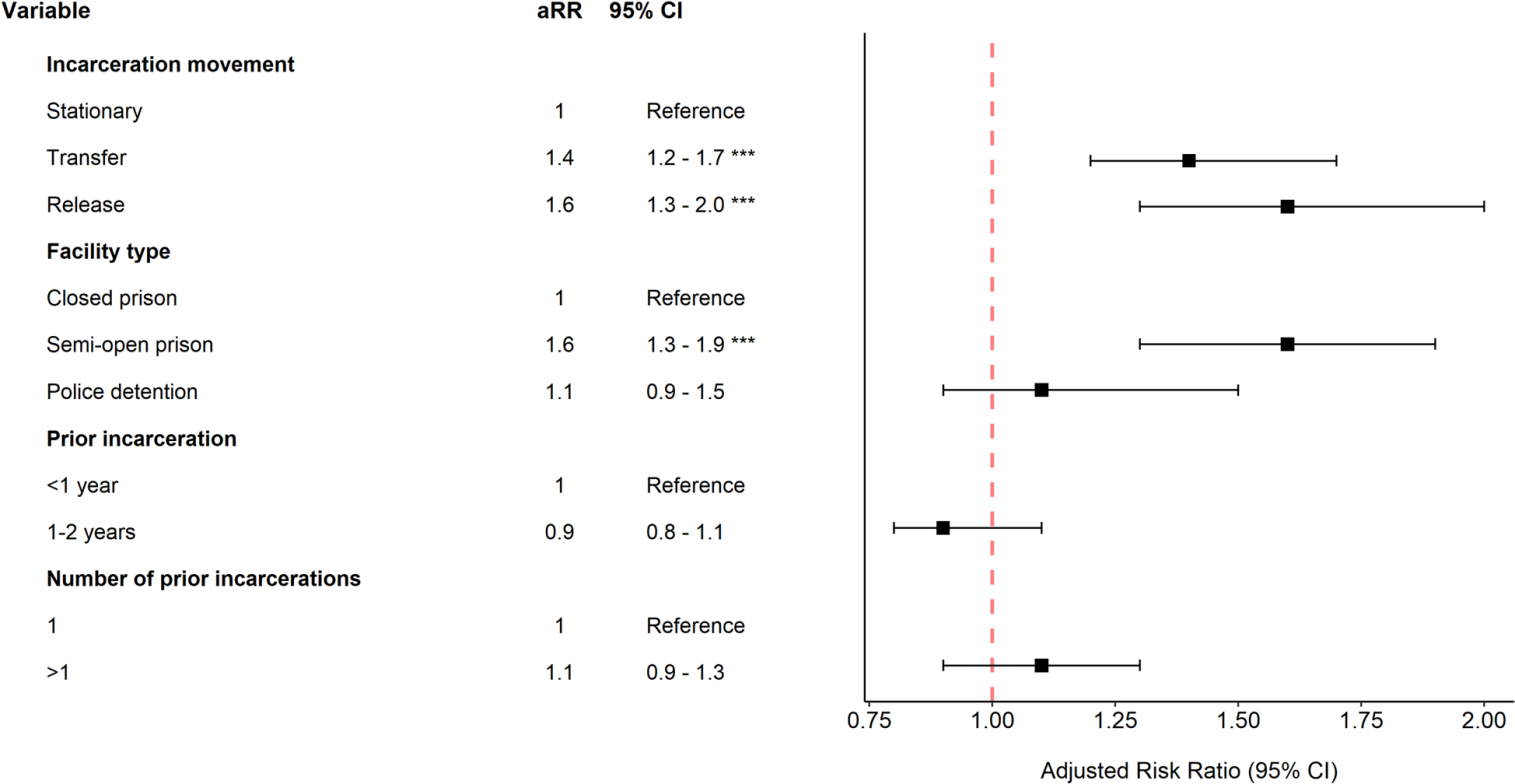
Adjusted relative risks associated with unfavorable treatment outcomes within 8 months of notification (carceral variables). Adjusted relative risks of unfavorable treatment outcomes evaluated 8 months after date of notification for incarceration-related variables. Unfavorable outcomes refer to all outcomes other than treatment success. The duration of previous incarceration and number of prior arrests were recorded in the 2 years preceding the date of notification. Facility type refers to the facility at the time of TB notification. Adjusted relative risks for date of notification can be found in Supplementary material 2: Table S5. Adjusted relative risks for demographic and clinical characteristics can be found in Supplementary material 2: Fig. S3. The model in this figure was adjusted for the following variables: incarceration movement, facility type, prior incarceration (years), number of prior incarcerations, sex, age, race, education, alcohol use disorder, mental illness, and year of notification

After adjusting for potential confounders, transfer between carceral facilities during TB treatment was associated with a 1.4-fold (95% *CI*: 1.2 to 1.7) increased risk of unfavorable treatment outcomes compared to the stationary group (Fig. 2). For individuals released from incarceration during treatment, the covariate-adjusted risk of unfavorable treatment outcomes was 1.6 (95% *CI*: 1.3 to 2.0) times as high as individuals in the stationary group. Results were similar in a sensitivity analysis excluding individuals who died from causes other than TB within 6 months following treatment initiation (Supplementary material 2: Table S6).

Beyond carceral movements, residing in a semi-open prison at the time of treatment initiation was associated with an increased risk of unfavorable treatment outcomes (adjustive relative risk [aRR]: 1.6, 95% *CI*: 1.3 to 1.9) (Fig. 2). However, this association did not remain after adjusting for individual carceral facility units in the regression (Supplementary material 2: Table S6). Additionally, a higher education level was associated with a significantly reduced risk of unfavorable treatment outcomes (*aRR*: 0.5, 95% *CI*: 0.3 to 0.9) (Supplementary material 2: Fig. S3).

### Treatment outcomes based on time of release

**Fig. 3:**
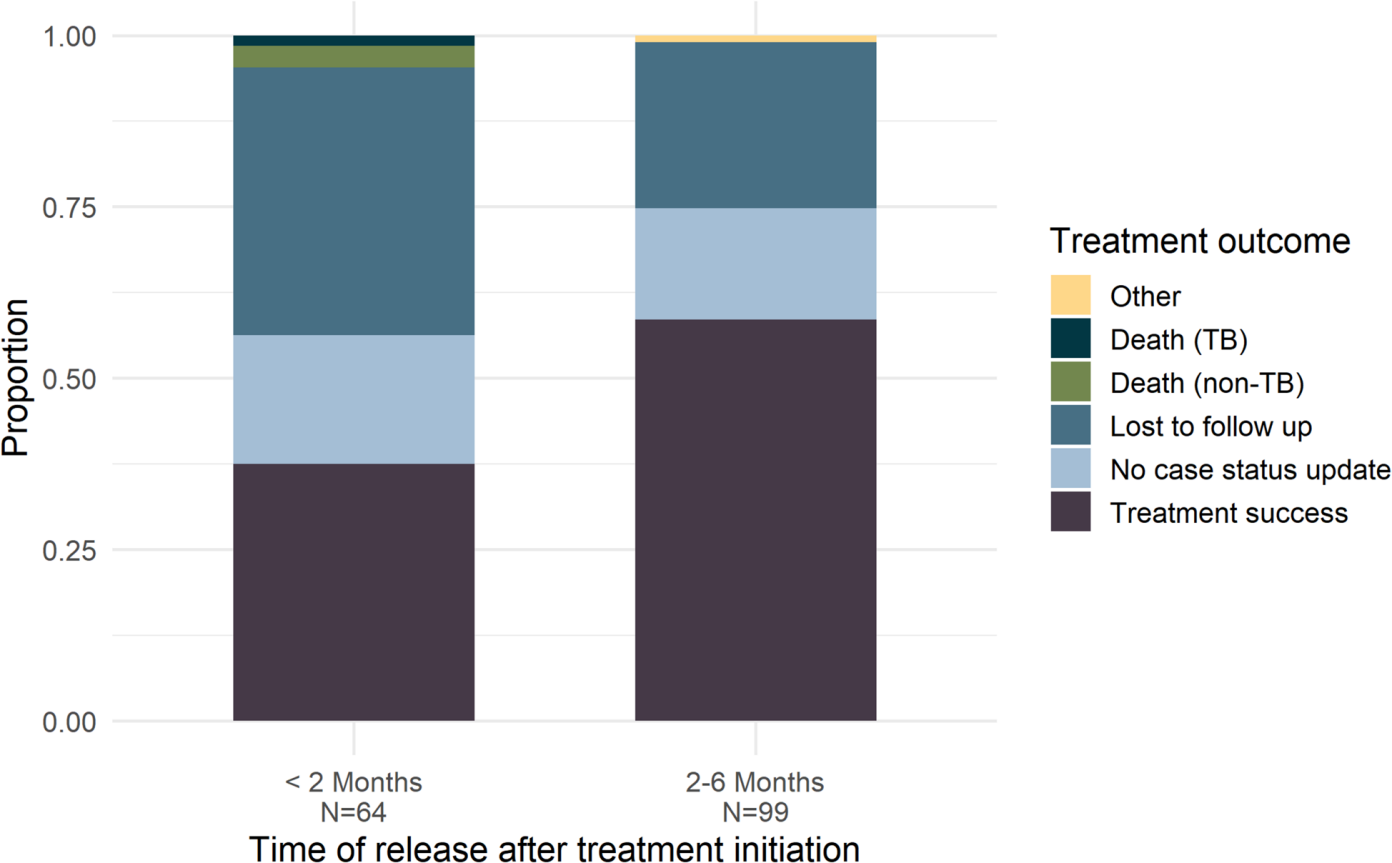
Tuberculosis treatment outcomes among individuals released from prison during treatment, stratified by time of release. Bar plots reporting treatment outcomes of individuals released less than 2 months or 2 months or more from the date of notification. No case status update includes individuals without any updates to their status in the TB database following treatment initiation, as well as individuals who were transferred to new health facilities/discontinued treatment, restarted treatment, and had no subsequent updates to their case status. Other refers to individuals who experienced change of diagnosis/regimen or were diagnosed with DR-TB

Among individuals in the released group, 39.2% were released less than 2 months after treatment initiation, during the intensive phase of TB treatment (Supplementary material 2: Fig. S4). The treatment success rate within 8 months was significantly lower among individuals released less than 2 months after initiating TB treatment (37.5%) compared to those who were released two or more months into treatment (58.5%) (*p*-value < 0.05) (Fig. 3). Additionally, the proportion of individuals lost to follow-up was 39.0% among those released less than 2 months after treatment initiation, compared to 24.2% among those released 2 months or later.

Release from incarceration within the first 2 months of TB treatment was associated with more than twice the risk of unfavorable treatment outcomes, compared to those who were stationary (*aRR* 2.1, 95% *CI*: 1.6 to 2.7) (Supplementary material 2: Table S7). For individuals released 2 months or more following treatment initiation, the risk of unfavorable treatment outcomes was 1.4 (95% *CI*: 1.0 to 1.8) times that of the stationary group.

## Discussion

Timely diagnosis and treatment completion are essential to reduce morbidity and mortality among TB patients and reduce onward transmission, particularly in high-risk environments like prisons. However, in settings with limited prison healthcare resources and barriers to coordination of care between prisons and communities, carceral facility transfers and release from incarceration may disrupt continuity of care. In this study, we linked incarceration and TB databases to determine the effect of carceral movements on TB treatment outcomes in a Brazilian state. Our findings revealed that approximately one in three PDL are transferred and/or released from the facility where they initiated treatment during the 6-month TB treatment period. Both releases and transfers were associated with increased risk of unfavorable treatment outcomes, after adjusting for covariates. Individuals released less than 2 months into treatment had particularly low rates of successful treatment completion. These findings underscore the need for strategies to enhance continuity of care for PDL with TB who are transferred between carceral facilities or released from incarceration during treatment.

Our findings provide novel insights into barriers to treatment completion among PDL in Brazil, identifying carceral movements as a major risk factor for unfavorable treatment outcomes in this population. Previous literature in the field has indicated that PDL has better TB treatment outcomes compared to the general population [7–12]. Although this may be true for a subset of patients who remain in the same facility during treatment, incarceration is dynamic, and facility transfers and releases are common. To date, studies in Brazil have been unable to test the effect of such movements on TB treatment outcomes due to the lack of detailed incarceration data in TB notification databases. Our database linkage approach addresses these data limitations, enabling ascertainment of carceral movements during TB treatment and assessment of associated treatment outcomes.

Here, we find low TB treatment success rates among individuals who are transferred or released from incarceration during treatment, comparable to those of other highly vulnerable groups including people living with HIV, people who are unhoused, and people who use drugs [33–37]. Moreover, the treatment success rate among all PDL in our cohort, regardless of carceral movement group, was lower than in previous studies from Brazil which report treatment success rates exceeding 80% among PDL [7, 10]. This discrepancy may stem from differences in the inclusion or classification of missing outcomes: while many prior studies exclude individuals without reported outcomes, we classify the lack of status update in the TB database as an unfavorable outcome. Addressing incomplete or delayed reporting in administrative databases is essential to improve monitoring and evaluation of TB treatment outcomes.

Our study has several limitations. Missingness within variables in the TB database (SINAN) prevented us from including several covariates in our analysis, such as smoking or drug use, which have been found to be associated with TB treatment outcomes [38–40]. Furthermore, there may have been residual confounding by other socioeconomic or incarceration-related covariates that were not available in our data. There may also be underreporting of clinical variables such as alcohol use disorder and mental illness. We found a nonsignificant association between mental illness and treatment success, likely due to the low prevalence of diagnosed mental illness within our cohort. Further studies are needed to assess this relationship. Additionally, there may be changes in the reporting of variables over time. We adjust for the year of notification in our model to help address these potential differences. Our study does not include final outcomes for individuals diagnosed with DR-TB. While MDR-TB prevalence is relatively low in Brazil (less than 3% for new cases) [41], further research is needed to evaluate DR-TB treatment outcomes for individuals experiencing carceral movements. Our analysis may be subject to survivorship bias, as individuals in the transferred or released groups had to survive long enough to experience carceral movements. If survival is associated with treatment success, this could bias our results toward the null, leading us to underestimate the relative risk of unfavorable treatment outcomes associated with carceral movements. However, the low proportion of deaths in our cohort suggests minimal impact. Moreover, in our sensitivity analysis excluding individuals with non-TB deaths within 6 months of treatment initiation, the estimates of relative risk associated with carceral movements remained largely unchanged (Supplementary material 2: Table S6). Another limitation is that individuals categorized as released in our analysis may have previously experienced transfers that contributed to treatment disruptions. We did not differentiate between individuals released directly from incarceration to the community and those who experienced one or more transfers prior to release. We also do not differentiate between individuals released following the end of their sentence and those who escaped from incarceration. Additionally, we were unable to obtain treatment outcome data for individuals who moved outside the state of Mato Grosso do Sul during the follow-up period or individuals who linked to care in the community under a different name. To address this uncertainty, we perform a sensitivity analysis in Supplementary material 2: Text S3. Even after accounting for potential out-of-state migration and limitations in record linkage, significant differences in treatment outcomes persist among those who experienced carceral movements and those who remained stationary. Finally, the results of this study may not generalize to other states or countries.

Our findings highlight the urgent need for comprehensive strategies to improve continuity of care for individuals diagnosed with TB who are transitioning within and out of the carceral system. Ensuring timely transfer of medical records when individuals move to different carceral facilities or communities is critical, especially for individuals transferred or released early during treatment. Currently, when people are released from incarceration, healthcare professionals receive no advance notice. In most regions in Brazil, there is no active system in place to locate people released from incarceration, nor are there designated healthcare facilities for this population. Additionally, there is no standardized process for patient transfers in carceral settings. Most correctional facilities do not maintain electronic health records for TB, leaving healthcare providers to rely on the notification system (SINAN) to access clinical history of persons with TB. Referrals to community health care clinics, case management services, and discharge planning may play pivotal roles in facilitating continuity of care post-release [42, 43]. In addition to improving system-level coordination, efforts to support individuals’ basic needs and social reintegration are essential. Individuals who are released from incarceration may face social instability, marginalization, and stigma, all of which can hinder care-seeking and access [44–47]. In the present study, we did not distinguish between people who were released versus those who escaped from incarceration, but the latter may experience heightened barriers to accessing healthcare. Further work is needed to understand these challenges and develop strategies to ensure all individuals, regardless of legal status or incarceration history, are supported to access healthcare services without fear of stigma or punishment.

## Conclusions

Our study found that both releases and transfers from carceral facilities were associated with increased risk of unfavorable treatment outcomes. Furthermore, individuals released earlier within their treatment regimen had higher loss to follow-up rates. Our results highlight the need for strategies to ensure continuity of care for people with TB who are impacted by incarceration. These strategies can have broader implications for continuity of care after incarceration for health conditions beyond TB, a topic that remains poorly understood in Brazil and many other low- and middle-income countries [48–51].

## Supplementary Information

Supplementary material 1. Native Language Abstract This file contains a Portuguese translation of the abstract.

Supplementary material 2. This file contains additional information on the methodology of our study as well as supplementary figures and tables. Text S1 Data deduplication/linkage and cohort construction details. Text S2 Outcome Interpretations. Text S3 Sensitivity Analysis. Table S1. Regression covariate missingness and imputation error. Table S2. Date of notification for incarceration cohort. Table S3. Carceral facility units for incarceration cohort. Table S4. TB treatment outcomes two years following treatment initiation. Table S5. Adjusted relative risk associated with unfavorable treatment outcomes 8 months post date of notification (extended table). Table S6. Adjusted relative risk associated with unfavorable treatment outcomes 8 months post date of notification (sensitivity analyses). Table S7. Adjusted relative risk associated with unfavorable treatment outcomes 8 months post date of notification with released individuals stratified by timing of release. Fig. S1 SIGO and SINAN matching criteria. Fig. S2 Carceral movements by facility type. Fig. S3 Adjusted relative risks associated with unfavorable treatment outcomes within 8 months of notification (demographic and clinical variables). Fig. S4 Distribution of time spent incarcerated from diagnosis to initial release. Fig. S5 Distribution of time spent incarcerated from diagnosis to initial transfer.

## Data Availability

This study utilized datasets provided by state agencies in Mato Grosso do Sul, Brazil, and we are not authorized to share them directly. The incarceration database was provided by Agência Estadual de Administração do Sistema Penitenciário (AGEPEN, [https://www.agepen.ms.gov.br/](https:/www.agepen.ms.gov.br)). The tuberculosis and mortality databases were provided by the Secretaria de Estado de Saúde (https://www.saude.ms.gov.br/).

## Abbreviations

TB: Tuberculosis
PDL: Persons deprived of liberty
aRR: Adjusted relative risk
SINAN: Sistema de Informação de Agravos de Notificação
SIGO: Sistema Integrado de Gestão Operacional
SIM: Sistema de Informações Sobre Mortalidade
DR-TB: Drug-resistant tuberculosis
