# Supplementary material for: Tuberculosis treatment outcomes after transfer or release from incarceration: a retrospective cohort study from Brazil
Source: BMC Glob Public Health. 2025 Oct 21;3:93. doi: 10.1186/s44263-025-00210-5 (PMC12541975; doi:10.1186/s44263-025-00210-5)
Supplement: Supplementary file 1 — Supplementary material 1. Native Language Abstract This file contains a Portuguese translation of the abstract. [file 44263_2025_210_MOESM1_ESM.pdf]

Additional file 1: Native Language Abstract

This translation in Portuguese was submitted by the authors, and we reproduce it as supplied. It has not been peer reviewed. Our editorial processes have only been applied to the original abstract in English, which should serve as reference for this article. This translated abstract is published under the same license as the article.

Contexto

A tuberculose (TB) afeta desproporcionalmente as pessoas privadas de liberdade (PPL). Estudos anteriores mostraram maiores taxas de conclusão do tratamento da TB entre PPL em comparação com a população geral. No entanto, pouco se sabe sobre como os movimentos relacionados ao encarceramento, como transferências entre unidades ou libertações para a comunidade, afetam os desfechos do tratamento da TB.

Métodos

Vinculamos dados individuais de encarceramento com dados de notificações de TB do Sistema de Informação de Agravos de Notificação (SINAN) para o estado brasileiro de Mato Grosso do Sul, entre janeiro de 2006 e dezembro de 2018. Construímos uma coorte de PPL com diagnóstico novo de TB sensível a drogas e que iniciaram tratamento. Comparamos os desfechos do tratamento entre indivíduos que permaneceram na mesma unidade prisional e aqueles que foram transferidos para outras unidades ou libertados durante o tratamento. Calculamos o risco relativo ajustado (RRa) de desfechos desfavoráveis do tratamento, controlando por covariáveis, para indivíduos transferidos ou libertados durante o tratamento.

Resultados

Identificamos 1.261 PPL que iniciaram tratamento para TB. Desses indivíduos, 842 (66,8%) permaneceram na mesma unidade prisional, 256 (20,3%) foram transferidos para outras unidades e 163 (12,9%) foram libertados para a comunidade durante o tratamento. Entre aqueles que permaneceram na mesma unidade, 72,9% (614/842) concluíram o tratamento com sucesso em até oito meses após o início. Em contraste, apenas 61,7% (158/256) dos transferidos e 50,3% (82/163) dos libertados alcançaram sucesso no tratamento da TB em até oito meses. Após ajuste pelas covariáveis, o risco de desfechos desfavoráveis do

tratamento foi 1,4 (IC 95%: 1,2 a 1,7) vezes maior para indivíduos transferidos para outras unidades e 1,6 (IC 95%: 1,3 a 2,0) vezes maior para indivíduos libertados, em comparação com aqueles que permaneceram na mesma unidade durante o tratamento. Para indivíduos libertados com menos de dois meses de tratamento, o risco de desfechos desfavoráveis foi duas vezes maior (RRa: 2,1; IC 95%: 1,6 – 2,7).

Conclusões

As transferências entre unidades e as libertações do encarceramento são comuns e podem constituir barreiras à conclusão do tratamento da TB. Estratégias para garantir a continuidade do cuidado entre unidades prisionais e entre o sistema prisional e o sistema de saúde comunitário são urgentemente necessárias para melhorar os desfechos da TB entre indivíduos impactados pelo encarceramento.

Palavras-chave

Encarceramento, Prisões, Tuberculose, Tratamento, Cascata de cuidados, Vinculação ao tratamento, Continuidade do cuidado, Pessoas privadas de liberdade, Brasil
