# Supplementary material for: Tuberculosis treatment outcomes after transfer or release from incarceration: a retrospective cohort study from Brazil
Source: BMC Glob Public Health. 2025 Oct 21;3:93. doi: 10.1186/s44263-025-00210-5 (PMC12541975; doi:10.1186/s44263-025-00210-5)
Supplement: Supplementary file 2 — Supplementary material 2. This file contains additional information on the methodology of our study as well as supplementary figures and tables. Text S1 Data deduplication/linkage and cohort construction details. Text S2 Outcome Interpretations. Text S3 Sensitivity Analysis. Table S1. Regression covariate missingness and imputation error. Table S2. Date of notification for incarceration cohort. Table S3. Carceral facility units for incarceration cohort. Table S4. TB treatment outcomes two years following treatment initiation. Table S5. Adjusted relative risk associated with unfavorable treatment outcomes 8 months post date of notification (extended table). Table S6. Adjusted relative risk associated with unfavorable treatment outcomes 8 months post date of notification (sensitivity analyses). Table S7. Adjusted relative risk associated with unfavorable treatment outcomes 8 months post date of notification with released individuals stratified by timing of release. Fig. S1 SIGO and SINAN matching criteria. Fig. S2 Carceral movements by facility type. Fig. S3 Adjusted relative risks associated with unfavorable treatment outcomes within 8 months of notification (demographic and clinical variables). Fig. S4 Distribution of time spent incarcerated from diagnosis to initial release. Fig. S5 Distribution of time spent incarcerated from diagnosis to initial transfer. [file 44263_2025_210_MOESM2_ESM.pdf]

Additional file 2

Text S1 Data deduplication/linkage and cohort construction details

SIGO deduplication strategy details

We conducted internal record linkage within SIGO using three key identifiers: name, mother's name, and an internal identification number (RGI). We used three different identifiers to mitigate potential challenges in matching entries due to missingness in mother's names, typos in names or mother's names, or inconsistencies in the recording of RGI numbers. Record linkage was performed in three stages:

1. RGI-Based Matching: Records that had the same RGI were grouped together based on approximate string matching on names, with scores that exceeded specific thresholds.
2. Name-Based Matching Across RGIs: Records with the same names and mother's names but different RGIs were compared and consolidated based on defined thresholds.
3. Full Database Matching: All records in the database were compared against one another and consolidated based on defined thresholds.

To reduce the total number of string-matching comparisons, we only performed fuzzy string matching on pairs of records that met a low initial threshold of similarity. Specifically, we computed character-level 4-grams for all names which represent all contiguous 4-character length sequences within a name (eg. The name 'Miguel' yields the 4-gram sequence 'Migu, igue, guel'). We computed a 4-gram similarity score by calculating the proportion of shared substrings within the 4-grams of two names. If at least 70% of substrings in both of the names were shared, then we proceeded to perform fuzzy string matching.

All thresholds were determined through manual review of records with the same RGI. We identified records that belonged to the same individuals and set thresholds accordingly.

We utilized three algorithms for fuzzy string matching:

1. Levenshtein Distance: This metric was applied to compute similarity scores for names and mother's names.
2. Space Score: A modified Levenshtein Distance that is computed by obtaining the Levenshtein Distance between two names after removing all white spaces in the names. We incorporated this score into our matching process to address inconsistencies in the spacing of names in the SIGO database.

3. Partial Matching Score: This score is designed to handle missing portions of names such as a missing middle name within a record. This score tokenizes the shorter of two names using white space delimiters. It compares each token to all adjacent substrings of the same length in the longer name. The maximum Levenshtein distance for each token compared over all substrings is stored, and these scores are averaged to generate the final partial matching score.

The space score and partial matching score were only computed if the Levenshtein Distance between two names were sufficiently high.

SINAN deduplication strategy details

To deduplicate SINAN entries, we used the 4-gram approach and the string-matching functions previously used in the SIGO deduplication process. We additionally incorporated date of birth comparisons between records. We set thresholds after manually examining entries with identical names and mother's names but different dates of birth, as well as entries with identical dates of birth but differing names and mother's names.

SIGO and SINAN database linkage approach

To link entries between SIGO and SINAN, we compared names and mother's names using the same matching functions used in the deduplication processes of the databases. We similarly eliminated comparisons by utilizing 4-grams. We determined thresholds for linkage by examining the sensitivity and specificity over a range of values. To calculate sensitivity, we constructed a true positive set of entries in SINAN that we expected to match to entries in SIGO. This true positive set consisted of individuals who were recorded as being in custody (using the POP_LIBER and INSTITUCIO variables) in SINAN in 2012 and later, during which reporting of incarceration status improved in SINAN. To calculate specificity, we similarly constructed a true negative set of entries that should not match to SIGO records. This true negative set included individuals aged 12 and under in SINAN as of December 31st 2018 (the final date for which SIGO data is available in our study), as individuals aged 12 and under would not be in custody. Our matching process yielded a sensitivity of 90%, a specificity of 95%, and positive predictive value of 98%.

SINAN and SIGO cohort filtering

After matching records between SINAN and SIGO, we obtained 9,172 potential matches. Given imperfect specificity of our matching algorithm, we performed additional filtering to remove false positives. We excluded 27 matches where individuals had reported carceral movements in SIGO prior to their date of birth in SINAN. We further eliminated 90 matches of individuals who were minors at their date of TB notification. Our matching approach between SINAN and SIGO entries allowed for entries in either database to be matched more than once. In order to eliminate matches for individuals matched to multiple records, we selected the match pair with the highest matching score. Note, in the case where multiple SIGO records were matched to the same SINAN record, if the carceral movement history indicated that two records belong to the same individual (eg. identical origins and destinations for recorded movements as well as a sequence of dates that aligns for both records), we consolidated these matches. Following this additional filtering, we obtained a sample of 5,637 individuals matched across SINAN and SIGO. We then subset this sample to only include 1,261 PDL who were newly diagnosed with TB and initiated TB treatment between January 1st,2006 and June 30th,2018. This cohort excludes individuals with DR-TB upon diagnosis as well as individuals who had changes to their diagnosis or treatment regimen during the follow-up period.

SIGO, SINAN, and SIM database linkage

To link entries from our SIGO-SINAN cohort with SIM, we applied the 4-gram comparison strategy used previously and compared names, mother's names, and dates of birth. For each entry in our cohort, matching was performed using the information within the SINAN database as it exhibited less missingness and fewer inconsistencies compared to SIGO.

We relied on deaths reported in both SINAN and SIM for recording outcomes in our cohort. We classified deaths as being TB-related and non-TB related. For records where at least one record indicated that the death was TB-related, we reported the death as TB-related. For discrepancies within the date of deaths, we used the earliest date in either system to address delays in reporting. All discrepancies in dates of death were of less than one month. At eight months following date of notification, six deaths were reported in SINAN that were not reported in SIM. 11 deaths were reported in SIM that were not recorded in SINAN. At two years following date of notification, nine deaths were reported in SINAN that were not present in SIM and 29 deaths were reported in SIM that were not present in SINAN.

SIGO, SINAN, and SIM cohort filtering

We retrieved 136 unique matches between SINAN and SIM. 114 of these matches had deaths within our study's observation period. We manually removed 4 false positive matches that included carceral movements or new TB cases reported after date of death in SIM.

Cohort Inclusion and Exclusion Criteria Details

We excluded individuals who had drug resistant TB upon diagnosis from our analysis. Those who developed drug resistant TB during treatment have DR-TB recorded as their final outcome as these patients are moved to a different TB database and we no longer have access to their outcomes.

We additionally excluded individuals with changes in diagnosis or treatment regimen.

In prisons in Mato Grosso do Sul, extrapulmonary TB cases are treated with the standard six-month regimen. Some pleural TB cases are treated for nine months while meningitis TB and bone TB may be treated for 12=month regimens. However, in our cohort, those who had pleural, meningitis, or bone TB had outcomes reported by eight months and are included within our analysis.

Group and Outcome Ascertainment

Individuals are classified into three carceral movement categories based on their most recent movement prior to experiencing a terminal outcome within six months of their date of notification. Terminal outcomes are defined as events that determine treatment success within the observation period. For the purpose of our analysis, we assume TB treatment requires six months for completion. The observation periods and corresponding terminal outcomes are listed below:

8-month observation period:

1. Lost to follow up with no resumption of treatment within 2 months
2. Treatment Success
3. Death
4. DR-TB

2-year observation period:

1. Treatment Success
2. Death
3. DR-TB

Text S2 Outcome Interpretations

We consider no case update in SINAN at 8 months as an unfavorable outcome because it reflects care disengagement and a lack of timely follow-up and case closure according to standard notification guidelines. All individuals with no case status update at 8 months had their outcome resolved by 2 years following notification (Additional file 2:Table S4). Only one individual of all three incarceration groups who had no case status update (primary) at 8 months was reported to have treatment success by 2 years.

Of note, no case update is to be distinguished from an unknown outcome. Unknown outcomes occur when a health provider inputs the closure status as "N/A". We also consider this an unfavorable outcome for similar reasons as above. The proportion of individuals with outcomes that are unknown in SINAN at 8 months post date of notification is similar across the three incarceration groups (Table 2). Patients who have not completed treatment successfully by the end of the follow-up period will have their most recent outcome reported as their final outcome. In the 8-month analysis, it is possible that an individual has no outcomes reported. In this case they will have "no case update" reported as their outcome.

Text S3 Sensitivity Analysis

Some individuals in our cohort who were released from incarceration and classified as having adverse treatment outcomes may have relocated out of state or used a different name in the community after release, precluding record linkage and true outcome ascertainment. We conducted a sensitivity analysis to examine the impact this uncertainty in record linkage may have on our results. We first identify individuals in the cohort who were released to the community within 8 months after notification and met any of the following criteria:

Those with unknown outcomes at 8 months

Those with an 8-month outcome of “referred to a different health facility” and either no changes to the outcome at 2 years or an unknown outcome at 2 years

People with no case status updates at 8 months (either primary or after resuming treatment post-treatment discontinuation/referral to different health facility), and an outcome at 2 years of either “referred to different health facility” or unknown

We assume the treatment success rate for people who move out of the state or use a different community name is the same as the rest of the cohort.

We found a total of 58 (4.6%) individuals in the cohort who met these criteria, of whom between 0-100% may have moved out of state or linked to care in the community under a different name.

If we assume 100% of people in all incarceration groups who may have moved out of state or linked to care in the community under a different name did so, the treatment success rates for the incarceration groups are as listed:

Stationary Group: 614/834 (73.6%)

Transferred Group: 158/237 (66.7%)

Released Group: 82/132 (62.1%)

(Chi-square p-value <.001)

Using data from Agência Estadual de Administração do Sistema Penitenciário (AGEPEN) on the two largest prisons in the state (EPJFC and IPCG), we found that 74% of PDL within the

state of Mato Grosso do Sul were born in the state. This suggests that most PDL have strong within-state ties and may be less likely to leave the state upon release. Moreover, while the SIGO data used in our analysis only includes information on prison transfers to facilities within the state of Mato Grosso do Sul, the AGEPEN data reports that fewer than 1% of prison transfers were out of state. We also note that the use of a “community name” that is different than the one used in the carceral system primarily occurs among PDL who escape from incarceration, who comprise a minority of people exiting the prison system. Therefore, we believe the true result is likely closer to that in our primary analysis. Moreover, individuals who relocate out-of-state or use a different name in the community after release may plausibly have greater barriers to treatment completion.

Table S1. Regression covariate missingness and imputation error

Regression covariate missingness and imputation results

Variables	Missingness	Imputation Error
Alcohol use disorder	24%	25%
Age	<1%	87 days
Mental Illness	23%	8%

Out-of-bag error is reported for each variable with missing values utilized in our regression analysis. For categorical variables, the imputation error is the proportion of false classifications. For continuous variables, the imputation error is the mean squared error. All regression covariates not included within this table, except for race and education, had 0% missingness. Due to high missingness for race and education, we did not utilize these imputed variables in our regression model and instead grouped missing values in the existing 'not reported' category within the data.

Table S2. Date of notification for incarceration cohort

Distribution of Year of Notification

Year of Notification	Stationary (N=842)	Transfer (N=256)	Release (N=163)
**2018**	76 (9.0%)	32 (12.5%)	16 (9.8%)
**2017**	103 (12.2%)	32 (12.5%)	18 (11.0%)
**2016**	129 (15.3%)	25 (9.8%)	18 (11.0%)
**2015**	79 (9.4%)	36 (14.1%)	15 (9.2%)
**2014**	85 (10.1%)	20 (7.8%)	13 (8.0%)
**2013**	95 (11.3%)	29 (11.3%)	15 (9.2%)
**2012**	83 (9.9%)	30 (11.7%)	16 (9.8%)
**2011**	54 (6.4%)	19 (7.4%)	14 (8.6%)
**2010**	43 (5.1%)	11 (4.3%)	4 (2.5%)
**2009**	40 (4.8%)	13 (5.1%)	20 (12.3%)
**2008**	36 (4.3%)	6 (2.3%)	9 (5.5%)
**2007**	13 (1.5%)	3 (1.2%)	4 (2.5%)
**2006**	6 (0.7%)	0 (0.0%)	1 (0.6%)

Table S3. Carceral facility units for incarceration cohort

Distribution of individuals who were diagnosed and initiated treatment in each carceral facility.

Carceral Facility	Stationary (N=842)	Transfer (N=256)	Release (N=163)
**EPJFC**	249 (29.6%)	82 (32.0%)	30 (18.4%)
**IPCG**	104 (12.4%)	46 (18.0%)	14 (8.6%)
**PED**	119 (14.1%)	22 (8.6%)	6 (3.7%)
**CPAIG**	34 (4.0%)	17 (6.6%)	16 (9.8%)
**PSMN**	32 (3.8%)	7 (2.7%)	7 (4.3%)
**EPMRSA-D**	22 (2.6%)	5 (2.0%)	14 (8.6%)
**EPC**	26 (3.1%)	8 (3.1%)	7 (4.3%)
**EPPAR**	22 (2.6%)	6 (2.3%)	2 (1.2%)
**EPRACA-CG**	14 (1.7%)	3 (1.2%)	12 (7.4%)
**PSM-TL**	17 (2.0%)	3 (1.2%)	4 (2.5%)
**PDIB**	16 (1.9%)	6 (2.3%)	2 (1.2%)
**CPA**	6 (0.7%)	7 (2.7%)	7 (4.3%)
**EPAM**	12 (1.4%)	4 (1.6%)	3 (1.8%)
**EPA**	8 (1.0%)	6 (2.3%)	2 (1.2%)
**Other**	161 (19.1%)	34 (13.3%)	37 (22.7%)

Carceral facilities within this table are the top 14 most populated within the cohort. All other facilities are grouped into 'other'. Percentages may not add up to 100 due to rounding.

Table S4. TB treatment outcomes two years following treatment initiation

TB treatment outcomes 2 years post date of notification

Treatment Outcome	Stationary Group (N=842)	Transfer Group (N=255)	Release Group (N=163)
**Treatment success**	626 (74.3%)	168 (65.9%)	89 (54.6%)
**No case status update (primary)**	0 (0.0%)	0 (0.0%)	0 (0.0%)
**No case status update (after resuming treatment post treatment discontinuation/referral to different health facility)**	0 (0.0%)	0 (0.0%)	0 (0.0%)
**Referred to different health facility with no follow-up record**	27 (3.2%)	31 (12.2%)	15 (9.2%)
**Treatment discontinuation with no follow-up record**	60 (7.1%)	23 (9.0%)	34 (20.9%)
**Referred to different health facility/treatment discontinuation and resumed with no case status update**	0 (0.0%)	0 (0.0%)	0 (0.0%)
**Death TB**	13 (1.5%)	1 (0.4%)	2 (1.2%)
**Death non-TB**	36 (4.3%)	4 (1.6%)	4 (2.5%)
**DR-TB**	1 (0.1%)	1 (0.4%)	1 (0.6%)
**Unknown**	79 (9.4%)	27 (10.6%)	18 (11.0%)

Carceral movement subgroups were determined at six months following treatment initiation and individuals may have experienced additional carceral movements before the end of the follow up period. Individuals included in the 8-month analysis who experienced changes in diagnosis or treatment regimen between 8 months and 2 years were excluded from the 2-year analysis. Percentages may not add up to 100 due to rounding.

Table S5. Adjusted relative risk associated with unfavorable treatment outcomes 8 months post date of notification (extended table)

Adjusted relative risk associated with unfavorable treatment outcomes (extended)

Characteristics	Categories	aRR	95% CI
**Year of Notification**	2017-2018	Reference	Reference
	2016	1.1	0.8-1.4
	2015	0.9	0.6-1.2
	2014	0.9	0.6-1.2
	2013	0.9	0.6-1.2
	2012	1.3	1.0-1.7
	2011	1.3	1.0-1.8
	2010	1.1	0.7-1.6
	2009	0.7	0.5-1.1
	2008	1.2	0.8-1.8
	2007	1.6	1.0-2.6

Includes relative risks of additional variables for the regression model used in the primary analysis (Figure 2 and Figure S3). The model in this figure was adjusted for the following variables: incarceration movement, facility type, prior incarceration (years), number of prior incarcerations, sex, age, race, education, alcohol use disorder, mental illness, and year of notification.

Table S6. Adjusted relative risk associated with unfavorable treatment outcomes 8 months post date of notification (sensitivity analyses)

Adjusted relative risk associated with unfavorable treatment outcomes (sensitivity analyses)

Characteristics	Categories	Model 1		Model 2	
		aRR	95% CI	aRR	95% CI
**Incarceration Movement**	Stationary	Reference	Reference	Reference	Reference
	Transfer	1.3	1.1-1.6***	1.5	1.2-1.8***
	Release	1.7	1.4-2.1***	1.7	1.4-2.2***
**Facility Type**	Closed prison	Reference	Reference	Reference	Reference
	Semi-open prison	1.1	0.6-1.8	1.6	1.3-2.0***
	Police detention	1.0	0.6-1.5	1.1	0.8-1.4

*Includes relative risks for variations of the regression model used in the primary analysis. Model 1 was adjusted for the following variables: incarceration movement, facility type, prior incarceration (years), number of prior incarcerations, sex, age, race, education, alcohol use disorder, mental illness, year of notification and additionally adjusts for individual carceral facility units (seen in Table S3). Model 2 was adjusted for the following variables: incarceration movement, facility type, prior incarceration (years), number of prior incarcerations, sex, age, race, education, alcohol use disorder, mental illness, and year of notification. Model 2 excludes individuals who died from non-TB deaths within the first sixth months after treatment initiation. *** refers to .01 <p value <=.001. ** refers to 0.05 < p value <=.01. * refers to p value <=.05.*

Table S7. Adjusted relative risk associated with unfavorable treatment outcomes 8 months post date of notification with released individuals stratified by timing of release

Adjusted relative risk associated with unfavorable treatment outcomes (with released group stratified by timing of release)

Characteristics	Categories	aRR	95% CI
**Incarceration Movement**	Stationary	Reference	Reference
	Transfer	1.4	1.2-1.7***
	Release (<2 months post-notification)	2.1	1.6-2.7***
	Release (2+ months post-notification)	1.4	1.0-1.8*
**Facility Type**	Closed prison	Reference	Reference
	Semi-open prison	1.6	1.3-1.9***
	Police detention	1.1	0.8-1.4

*Includes relative risks for regression model stratifying individuals who were released into two groups: those who were released less than two months after treatment initiation and those released two or more months following treatment initiation. The model in this figure was adjusted for the following variables: incarceration movement (stratified by time of release), facility type, prior incarceration (years), number of prior incarcerations, sex, age, race, education, alcohol use disorder, mental illness, and year of notification. *** refers to .01 <p value <=.001. ** refers to 0.05 < p value <=.01. * refers to p value <=.05.*

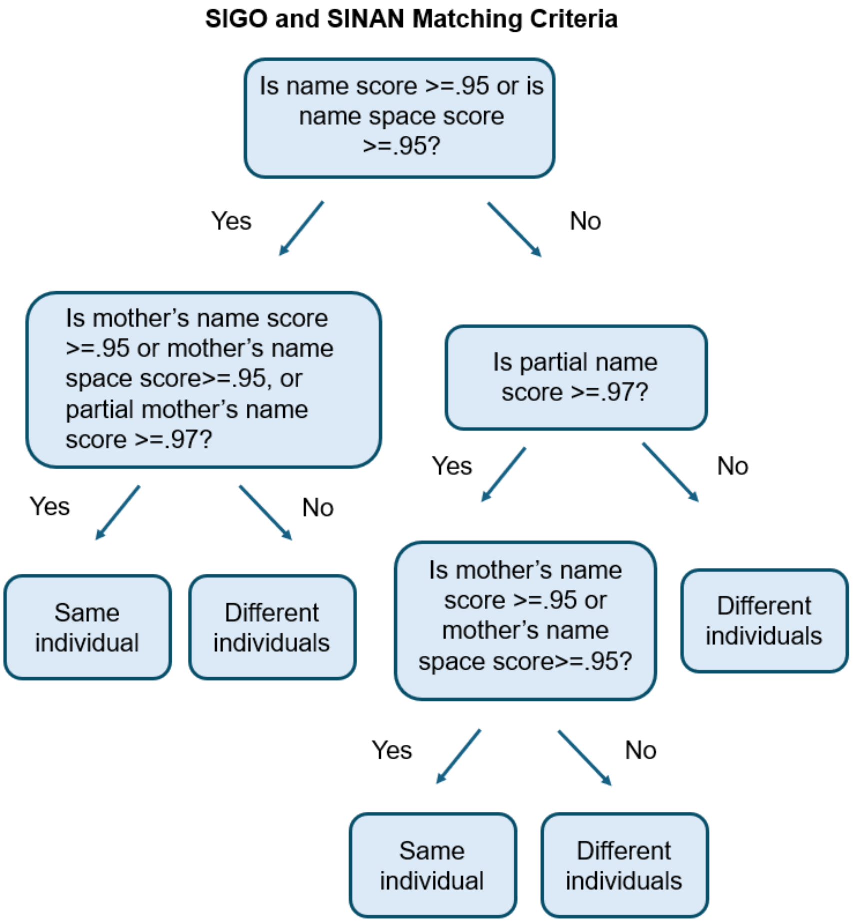

Fig. S1 SIGO and SINAN matching criteria

Decision tree depicting criteria used for identifying individuals between the SIGO and SINAN databases. String similarity was computed using functions described in Text S1.

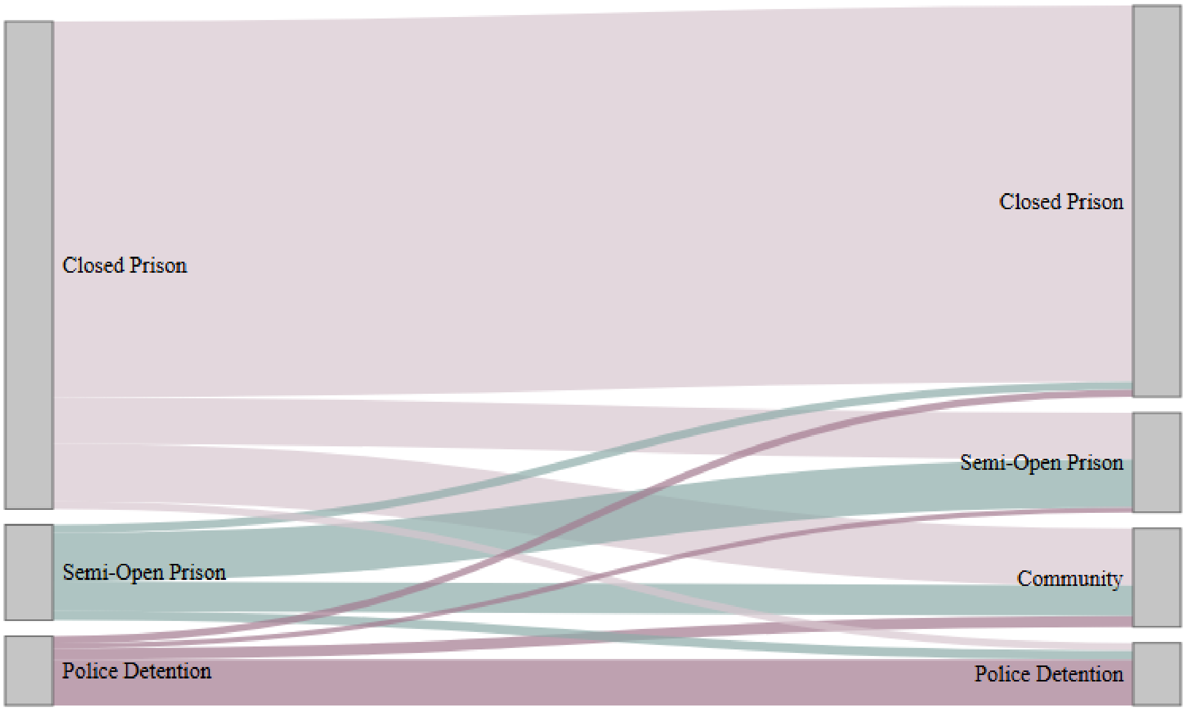

Fig. S2 Carceral movements by facility type

Sankey diagram showing carceral movements from the carceral facility location at the time of TB notification to the carceral facility location six months post-notification. Note that individuals may have experienced transfers between different locations of the same facility type (i.e., from one closed prison to another).

Variable	aRR	95% CI
**Sex**		
Male	1	Reference
Female	1.1	0.7 - 1.8
**Age**		
18-29	1	Reference
30-44	1	0.9 - 1.2
>45	1.1	0.8 - 1.4
**Race**		
White	1	Reference
Black	1	0.7 - 1.4
Asian	1.2	0.7 - 2.2
Brown	1.1	0.9 - 1.4
Indigenous	0.6	0.3 - 1.3
Not reported	1	0.8 - 1.3
**Education**		
Did not complete high school	1	Reference
Completed high school/higher education	0.5	0.3 - 0.9 *
Not reported	1	0.8 - 1.2
**Alcohol use disorder**		
No	1	Reference
Yes	1	0.8 - 1.2
**Mental illness**		
No	1	Reference
Yes	0.7	0.4 - 1.4

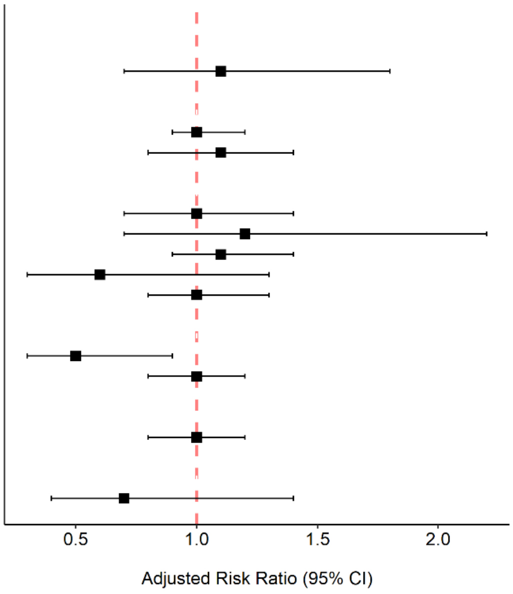

Fig. S3 Adjusted relative risks associated with unfavorable treatment outcomes within 8 months of notification (demographic and clinical variables)

Adjusted relative risks of unfavorable treatment outcomes evaluated eight months after date of notification for demographic and clinical variables. Unfavorable outcomes refer to all outcomes other than treatment success. The model in this figure was adjusted for the following variables: incarceration movement, facility type, prior incarceration (years), number of prior incarcerations, sex, age, race, education, alcohol use disorder, mental illness, and year of notification.

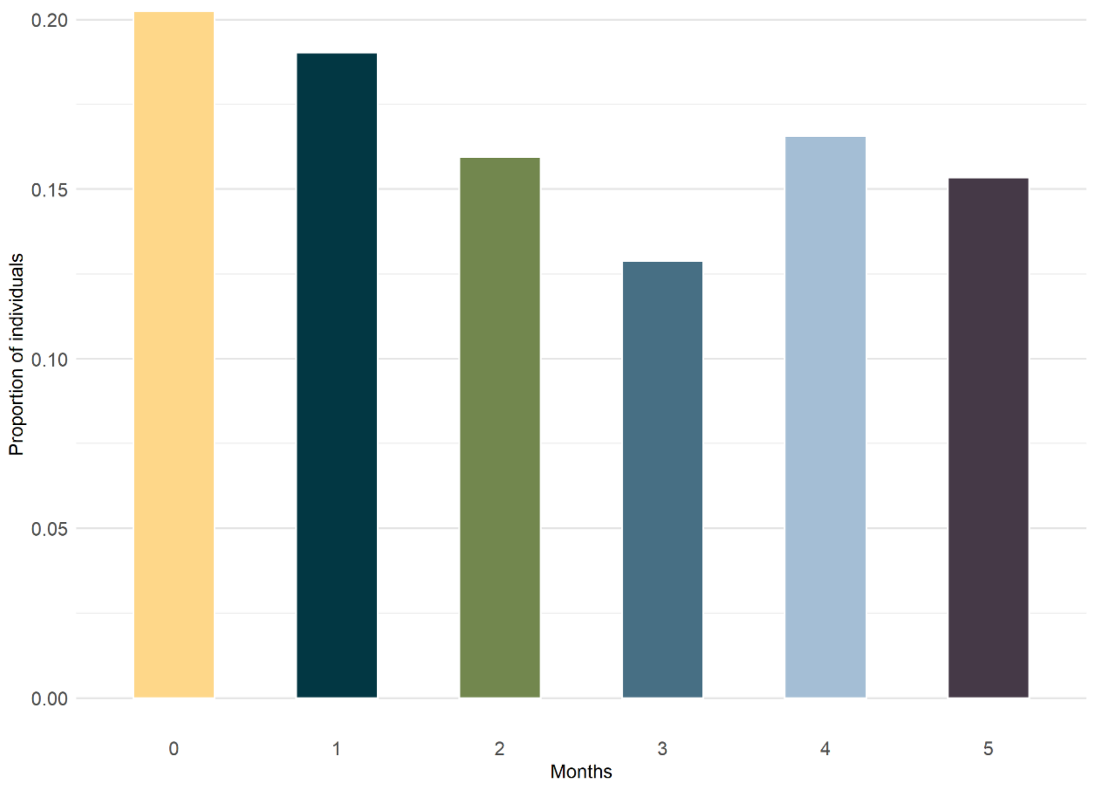

Fig. S4 Distribution of time spent incarcerated from diagnosis to initial release

Bar chart depicting the time in months individuals spent incarcerated after date of notification until initial release. This figure is comprised of individuals within the 'release' carceral movement category. The distribution of time individuals spent incarcerated prior to initial transfer can be found in (Additional file 2: Fig. S5).

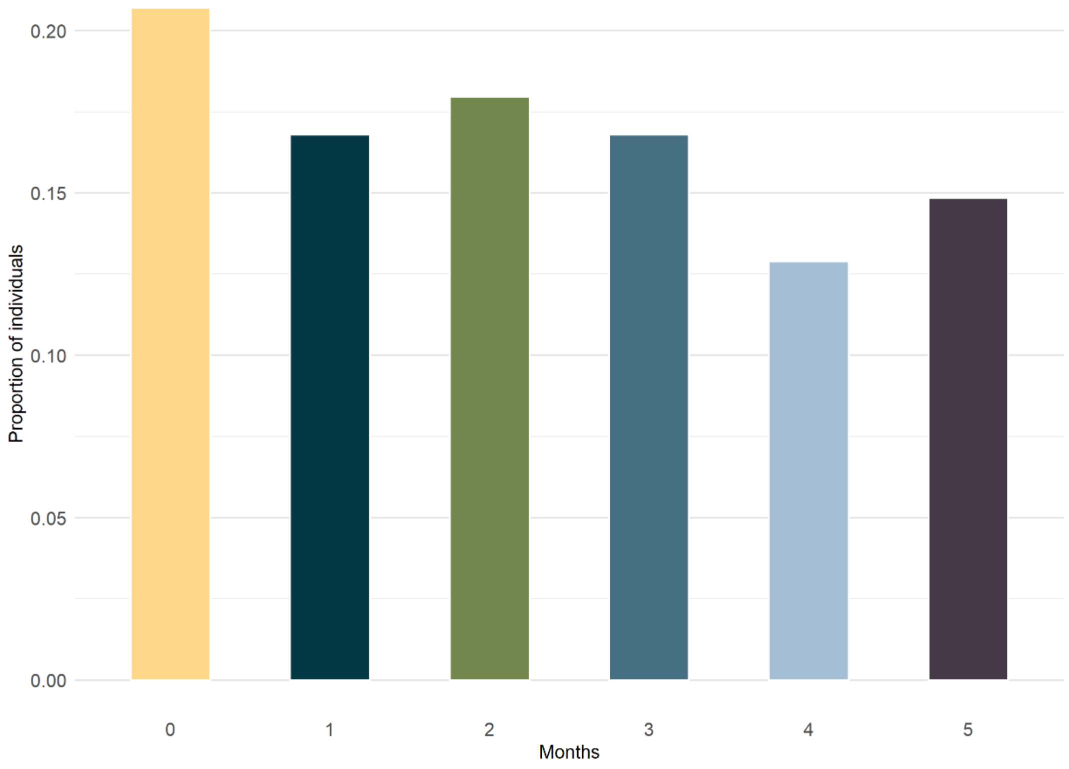

Fig. S5 Distribution of time spent incarcerated from diagnosis to initial transfer

Bar chart depicting the time in months individuals spent incarcerated after date of notification until initial transfer. This figure is comprised of individuals within the ‘transfer’ carceral movement category.
